# Evaluation of the Accuracy of 3D Printed Patient-Specific Brain Biopsy Guide Using 3D Volume Rendering Technique in Canine Cadavers

**DOI:** 10.3390/ani14213163

**Published:** 2024-11-04

**Authors:** Minseung Jeong, Jongchan Ko, Yong Yu, Suyoung Heo

**Affiliations:** College of Veterinary Medicine, Jeonbuk National University, Iksan 54596, Republic of Korea; chriszang@naver.com (M.J.); chan1207s@jbnu.ac.kr (J.K.); skyyong07@gmail.com (Y.Y.)

**Keywords:** brain biopsy, 3D printed guides, 3D volume rendering, needle placement accuracy

## Abstract

The objective of this study was to evaluate the accuracy of a CT-based, 3D-printed, patient-specific brain biopsy guide (3D-psBBG) through the application of a transfrontal approach in canine cadavers. A total of ten canine cadavers, with weights ranging from 4.36 to 14.4 kg, were subjected to preoperative CT scans to generate 3D skull models. Customized biopsy guides were created based on these models and manufactured using 3D printing technology. Twenty spinal needle insertions were performed, and the accuracy of needle placement was evaluated through both CT and 3D volume-rendering techniques. The mean needle placement error was 2.1 mm, with no significant differences observed between insertions targeting the fronto-olfactory and piriform lobes. The 3D volume-rendering method demonstrated superior accuracy compared to the CT method, with statistically significant differences in placement errors for both targets. The average time required for the design and manufacture of the guides was 249 min. These findings indicate the high accuracy and potential clinical application of CT-based 3D-psBBG for improving diagnostic outcomes in veterinary neurology.

## Simple Summary

Diagnosing brain diseases in animals presents significant challenges, particularly due to the difficulty of obtaining brain tissue samples with high precision. In this study, we developed a novel tool to assist veterinarians in performing brain biopsies more accurately in dogs. Customized guides were created through 3D printing using CT scans of the dogs’ skulls as a foundation to ensure precise needle placement. When tested on ten dog cadavers, the tool demonstrated a high level of accuracy with minimal placement errors. This innovation has the potential to improve the diagnosis and treatment of brain diseases in dogs, leading to better health outcomes for pets with neurological conditions. By enhancing the biopsy precision, this method can benefit both veterinarians and pet owners by providing more reliable diagnostic information.

## 1. Introduction

The reported occurrence rate of canine nervous system tumors is estimated at 14.5 cases per 100,000 dogs [1]. In clinical practice, approximately 90% of primary brain tumors in dogs comprise meningiomas (50%), gliomas (35%), and choroid plexus tumors (CPTs; 7%) [1,2,3,4,5]. Gliomas are the most common intra-axial brain tumors and are predominantly located within the fronto-olfactory, parietal, and temporal lobes of the brain [4,5,6].

Most intracranial lesions in dogs can be accurately detected using CT or magnetic resonance imaging(MRI) [7]. Despite the sensitivity of CT and MRI in defining brain lesions, their diagnostic specificity is constrained, particularly in differentiating between neoplastic, vascular, and inflammatory pathologies [8,9]. In a study involving dogs with brain lesions detected on MRI scans, researchers aimed to distinguish between gliomas and presumed cerebrovascular accidents. The findings revealed a notable discrepancy in the identification of diseases, with 10–47% of cerebrovascular accidents incorrectly classified as gliomas. Conversely, gliomas are misdiagnosed as infarctions in up to 12% of cases [10]. The imaging manifestations of these conditions often overlap considerably, which poses challenges in accurate diagnosis based solely on radiological findings [8,9]. Given that CT and MRI offer a limited range of potential diagnoses for identified lesions, brain biopsy has emerged as a crucial procedure, allowing for a definitive histological diagnosis [11].

Research into frame-based stereotactic brain biopsy and frameless image-guided neuronavigation (FLIGN) methods for canine brain biopsy has been conducted extensively, and their utility has been well-documented [12,13,14,15,16]. Recently, there has been an advancement in three-dimensional (3D) patient-specific printing technology in the field of veterinary neurology, and research on brain biopsy using 3D printing technology in dogs is increasing [7,17,18,19]. In these studies, 3D patient-specific guides (3DPSGs) were generated from MRI or CT scans to compare the deviation between the target point and the biopsy needle. The needle placement error ranged from 0.83 mm to 2.7 mm, showing high accuracy [7,19]. However, previous studies assumed target points in the caudate nucleus and piriform lobe, limiting access to the rostrotentorial approach. Moreover, they rely on virtual regions of interest (ROIs) without incorporating actual tumor models. Furthermore, these studies only compared target placement errors without providing detailed descriptions of the needle trajectories and actual positions [7,19].

This study aimed to evaluate the needle placement error and needle trajectory during brain biopsy using a CT-based 3D patient-specific brain biopsy guide (3D-psBBG). Furthermore, tumor models were inserted into the fronto-olfactory and piriform lobes to compare the method of confirming needle placement errors using CT imaging with the 3D volume-rendering method. The hypotheses were that (1) CT-based 3D-psBBG would show great accuracy and (2) the 3D volume-rendering method would be more precise than the CT method.

## 2. Materials and Methods

### 2.1. Cadaver Preparation

In this study, 10 canine cadavers were used. All dogs were sacrificed for reasons not related to the study. The mean body weight was 8.05 kg (median, 7.6; range, 4.3–14.4). The breeds included Jindo dogs (2), poodles (1), miniature schnauzers (1), and mixed breeds (6). The cadavers were stored at −20 °C and thawed at −4 °C for 72 h before imaging and surgery. This study was approved by the Institutional Animal Care and Use Committee of Jeonbuk National University (approval number: NON2024-075).

### 2.2. Imaging and Production of the Biopsy Guide

Preoperative CT scans were performed using a 16-slice helical CT scanner (Toshiba Alexion 16; Toshiba Medical System, Tochigi, Japan) with the following parameters: the field of view was set at 170 × 170 with a slice thickness of 0.5 mm in the transverse plane. The cadavers were placed in sternal recumbency, with the head as parallel as possible to the CT table. The acquired digital imaging and communications in medicine (DICOM) images were exported to Mimics Medical software (version 21.0; Materialize NV, Leuven, Belgium) to create stereolithography files. The skull was separated by threshold segmentation and the STL file of the head bone was exported to computer-aided design software (Fusion 360, Autodesk, San Rafael, CA).

**Figure 1:**
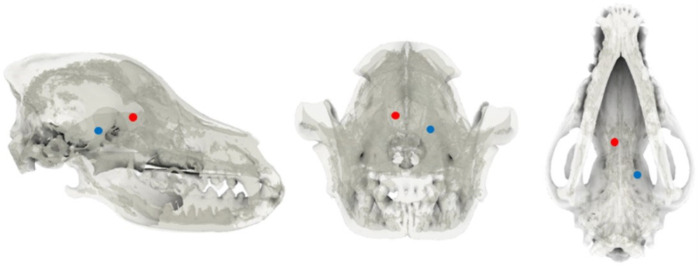
Tumor location selection. After reducing skull opacity, the sagittal, transverse, and dorsal planes were examined. The fronto-olfactory lobe region of interest (ROI) was identified and marked with a red sphere, while the piriform lobe ROI was delineated with a blue sphere.

The skull was adjusted to 10% transparency and realigned along the X, Y, and Z axes. In the dorsal plane, the internasal suture to the external sagittal crest was aligned along the X-axis, while in the transverse plane, the zygomatic processes of the frontal bone on both sides were horizontally positioned. The frontal sinus, frontal lobe, and olfactory lobe were identified in the sagittal plane, and the symmetrical regions were adjusted to overlap as closely as possible along the X-Z plane. To establish the target area and for the injection of the tumor model, spherical markers with a diameter of 1.5 mm were placed in the right fronto-olfactory and left piriform lobes to visualize the ROIs (Figure 1).

**Figure 2:**
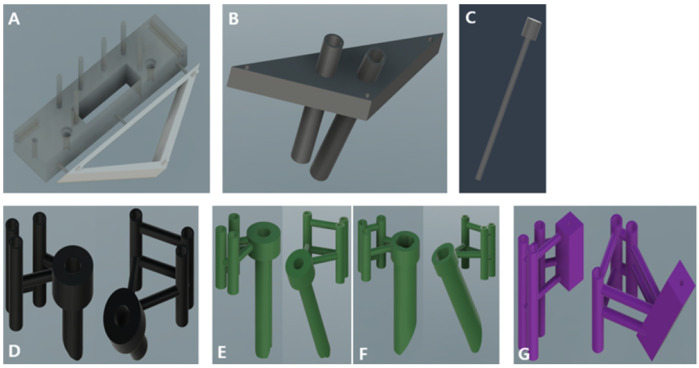
Guide configuration. (**A**) Main body, frontal body. (**B**) Tumor insertion unit. (**C**) Bar. (**D**) External burr units. (**E**) Internal burr unit 1. (**F**) Internal burr unit 2. (**G**) Needle insertion units.

Guides consisted of the main body, frontal body, tumor insertion unit, bar, burr units, and needle units (Figure 2). The first component, the main body, served as the core of the 3D guide, and the other parts of the guide were connected to perform the biopsy. Initially, a line was formed that connected the bilateral zygomatic processes of the frontal bone, and a rectangle was created caudally from this line including the frontal sinus in the dorsal plane. In the sagittal plane, extrusion was applied along the temporal line (orbitotemporal crest) to the highest point. To secure the guide to the skull, 2.0 mm cortical screw holes were created at the ends of the guide, ensuring that they did not extend beyond the line of the zygomatic processes of the frontal bone in a lag fashion. Subsequently, rectangular biopsy windows were used for instrument manipulation. Additionally, six cylindrical shapes with a diameter of 1.5 mm and a length of 15 mm were created for burr and needle-guide fixation. To assess the accuracy of compression after guide placement, two 1.5 mm holes parallel to the sagittal and transverse planes of the main body were drilled on each side, along with one 1.5 mm hole in the dorsal plane.

**Figure 3:**
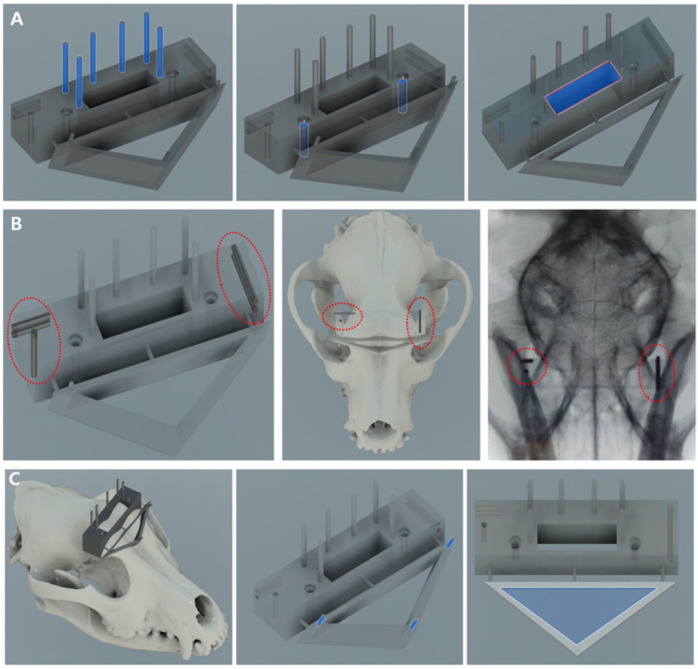
Attachment of the main body and frontal body to the skull. (**A**) The main body was a rectangular cuboid structure that featured a rectangular surgical window, six supports, two screw holes, and holes for K-wire insertion. (**B**) To evaluate the accuracy of compression following guide placement, two holes parallel to the sagittal and transverse planes, and one hole parallel to the dorsal plane, were created in the rectangular body. (**C**) The frontal body was a hollow triangular prism connected to the main body, equipped with three supports for the attachment of the tumor insertion unit.

The second component, the frontal body, served as a guide for the formation of a triangular bone flap through the transfrontal craniotomy. On both sides, a triangular surface was created that connected the zygomatic processes of the orbit to the junction of the nasal bone. In the sagittal plane, the triangular surface was extruded 10 mm in the rostroproximal direction at a 30° angle relative to the vertical line of the main body, and the area to perform the triangular-shaped osteotomy was cut. At each vertex of the triangular surface, three cylindrical shapes with a diameter of 1 mm for connection to the tumor insertion guide were extruded by 5 mm. Subsequently, cylindrical bridges were used to connect the main body (Figure 3).

**Figure 4:**
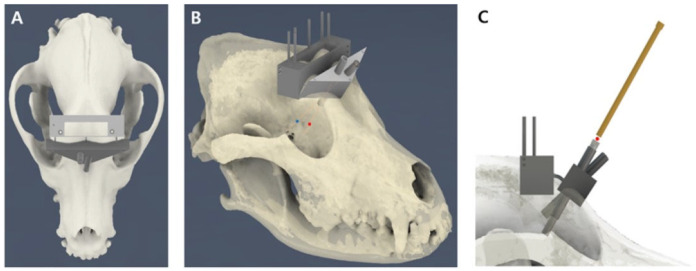
Tumor model injection. (**A**) Frontal body with the attached tumor insertion unit. (**B**) The red sphere indicates the target in the fronto-olfactory lobe, while the blue sphere signifies the target in the piriform lobe. The orange axis represents the line that connects the centers of each target to the center of the tumor insertion part. (**C**) The bar was used to insert the tumor model.

The third tumor insertion unit served as a guide for the insertion of the tumor model. It underwent a 5 mm extrusion parallel to the frontal body plane, leaving holes for cylindrical shapes with a diameter of 1 mm at each vertex of the triangular surface. The center point of each sphere, defined as the ROI, was set along with a point in the proximal plane of tumor insertion. An axis connecting these two points was established along which a cylindrical shape with an inner diameter of 4.3 mm and an outer diameter of 5.3 mm hollow inside) was aligned. Additionally, a cylindrical bar with a diameter of 4 mm was made to fit in the tumor model (Figure 4).

**Figure 5:**
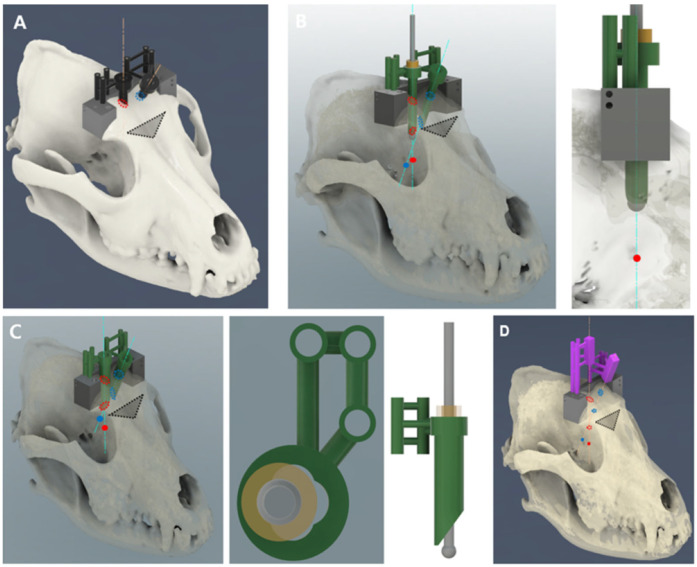
Needle placement procedure. (**A**) Portions of the frontal body and main body were removed and the black external burr unit was attached. (**B**) The green internal burr unit 1 was attached to penetrate the outer layer of the internal table of the frontal bone. The yellow ring indicates a stopper. (**C**) Unit 2 was inserted to penetrate the inner layer of the internal table of the frontal bone. (**D**) The purple needle guide was attached, and each axis represents the trajectory of the spinal needle insertion.

The fourth burr unit was designed to improve accuracy during round craniotomy. These were divided into external and internal parts, with the external burr unit used to penetrate the outer table of the frontal bone and the internal burr unit used for the inner table. For the external unit, when targeting the right fronto-olfactory lobe ROI in the sagittal plane, the axis was set perpendicular to the most proximal surface of the main body from the center of the tumor. A hollow cylinder with an inner diameter of 7.5 mm and an outer diameter of 8.5 mm was aligned along this axis, and an additional hollow cylinder with an inner diameter of 7.5 mm and outer diameter of 10 mm was added proximally by 5 mm to support the stopper (Figure 5A). For stabilization, a hollow cylinder was created that fit the 1.5 mm diameter rod of the main body, connecting to the portion where the burr unit was inserted. The internal unit was composed of two components: unit 1 was designed to penetrate the outer layer of the internal table of the frontal bone, and unit 2 was designed to penetrate the inner layer. Internal unit 1 was precisely designed to fit the internal table of the frontal bone and featured a hollow cylindrical shape with an inner diameter of 4 mm and an outer diameter of 5 mm. Internal unit 2, which was of similar design to internal unit 1, incorporated a four-leaf clover-shaped groove inside the cylinder to provide additional support to the burr (Figure 5B,C). In the case of the left piriform lobe ROI, an axis was set connecting a point on the frontal bone and the center of the tumor. The burr unit was designed to fit within the rectangular window of the main body. The subsequent process was identical to that used for the right burr unit.

The fifth needle section, where the spinal needle was inserted, utilized the axis established during the design of the burr unit. A circle with a diameter of 1.2 mm was formed within a 5 × 5 mm square from the main body and then extruded for 30 mm. To aid in visualization during spinal needle insertion, the guide was trimmed to 15 mm and aligned along the left and right axes. The support for this rectangular shape was identical to that of the burr unit (Figure 5D).

Finally, the main and frontal bodies used the cutting function of the skull to ensure precise attachment of the guide to the frontal bone. For the burr unit, cutting functions were used to match the external and internal tables of the frontal bone, resulting in the design of the external and internal burr guides. For the tumor insertion unit, the guide was tailored to precisely fit the internal table of the frontal bone. Designs were saved as STL files for 3D printing.

The biopsy guides were 3D-printed using a dental surgical guide resin (ZMD-1000B CLEAR-SG, Zenith, Daegu, Republic of Korea) with a resin 3D printer (Pixel One, Zerone, Gyeonggi, Republic of Korea). The guides were washed and dried for 30 min after printing and cured with UV light at a wavelength of 405 nm for 30 min (3DP-100S, CUBICON, Gyeonggi, Republic of Korea).

### 2.3. Surgical Procedure

The cadavers were placed in the sternal recumbent position. The head was raised by securing the cadaver with an immobilization mattress (VACCUMAT, Genia, Saint-Hilaire de Chaléons, France). The head was clipped from the occipital protuberance to the orbital protuberance and nasal bones. A midline incision was made from the caudal edge of the nasal bone to the external sagittal crest. Subcutaneous tissues including the periosteum were undermined and reflected laterally using periosteal elevators [20].

**Figure 6:**
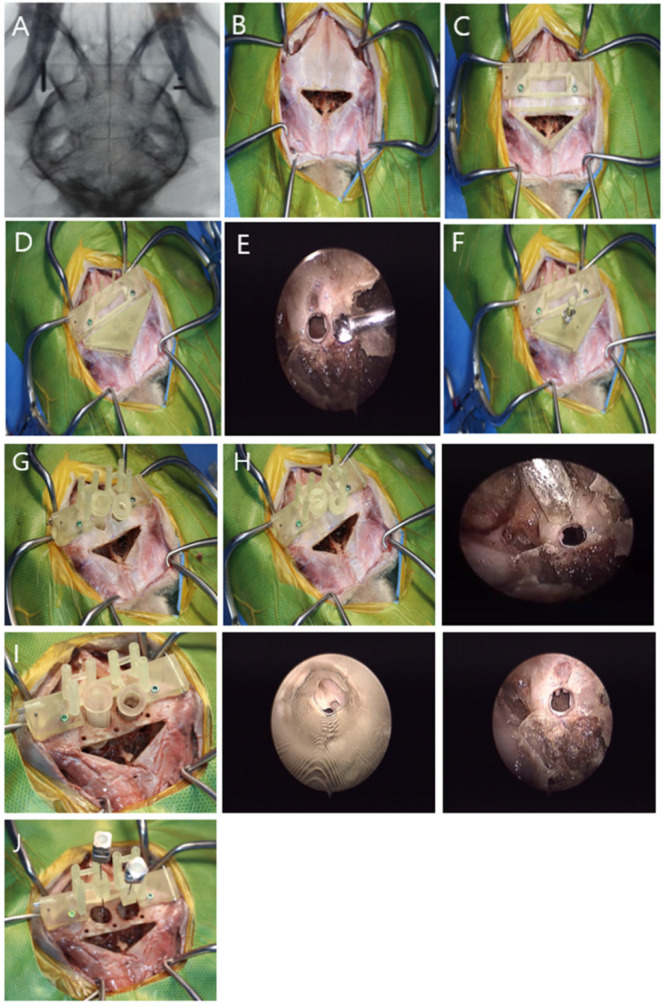
Surgical procedure for brain biopsy using a 3D-psBBG. (**A**) The alignment of the K-wire and the proper placement of the guide were confirmed by fluoroscopy. (**B**) A triangular craniotomy was performed to insert the tumor model. (**C**) The main body and the frontal body were mounted on the skull. (**D**) Placement of the tumor insertion unit. (**E**,**F**) After creating a hole for the tumor model, a 12-gauge spinal needle and bar were used to advance the tumor model to the planned target. (**G**) External burr units were mounted to create a hole in the outer frontal bone. (**H**) Attachment of the internal unit 1 guide. (**I**) Attachment of the internal unit 2 guide. (**J**) The 23-gauge spinal needles were in the needle insertion part on both sides.

The temporalis muscle was carefully dissected, starting from the zygomatic process of the frontal bone and extending caudally until the 3D-psBBG could be positioned on the skull. Subsequently, the 3D-BBG was attached to the skull, and intraoperative fluoroscopy (ZEN-2090 Pro, GENORAY Inc., Gyeonggi, Republic of Korea) was used to verify the appropriate placement of the guide by assessing the arrangement of the K-wires in the main body (Figure 6A). Once it was deemed appropriate, the triangular-shaped area was marked with a sterile skin marker (DeRoyal Industries, Powell, TN, USA). Subsequently, bone osteotomy was performed by inclining the oscillating saw at a 30° angle (Figure 6B). The flap was elevated dorsally and detached from the frontal sinus septum. After removing the frontal bone, the thin bone of the frontal sinus septum was removed using a rongeur. Drilling was performed using a 1.5/2.0 drill sleeve (ABLE Inc., Jeonbuk, Republic of Korea) and 1.5-mm drill bit (ABLE Inc.), and a stainless steel self-tapping 2.0 mm cortical screw (ABLE Inc.) was placed bicortically (Figure 6C).

A tumor insertion guide, designed precisely to fit the internal table of the frontal bone, was attached to the frontal body (Figure 6D). The craniectomy area was marked and removed using an 18-gauge needle. Bilateral holes were created using a 3 mm oval burr to accommodate the tumor model. If a defect was observed, the tumor insertion guide was reattached to the frontal body and a No. 12 spinal needle was inserted. Subsequently, a 1.5 mm diameter sphere, 3D-printed with resin (ZMD-1000B CLEAR-SG, Zenith, Daegu, Republic of Korea) and coated with iohexol (Omnipaque™ 300 mg/mL), was inserted into the hole using a bar to the calculated depth (Figure 6E,F).

Subsequently, parts of the frontal and main bodies were removed. An external burr unit was connected to the main body, and a 5-mm oval burr was used to perform a craniectomy on the frontal bone (Figure 6G). Once the space was created for the internal burr unit, it was connected to the main body, and a 2 mm oval burr with a stopper was used for further craniectomy (Figure 6H,I). Subsequently, the needle insertion unit was attached, and a 23-gauge spinal needle (HAKKO Co., Nagano, Japan) was inserted to the calculated depth from the center of the tumor to the midpoint of the most proximal surface of the needle insertion unit (Figure 6J). To verify the accuracy of the needle placement, a CT scan was performed. Finally, the triangular bone flap was sutured using 0 polydioxanone (PDS, Johnson and Johnson, New Brunswick, NJ, USA) through pre-drilled holes. A supplementary video explaining the detailed surgical technique is available in Supplementary Video S1 for clearer understanding.

### 2.4. Postoperative CT and 3D Volume-Rendering Analysis

**Figure 7:**
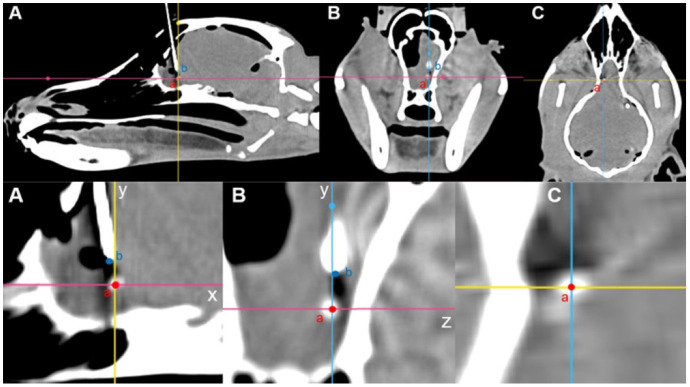
Calculation of needle placement error in the fronto-olfactory lobe after inserting a spinal needle, as evaluated by CT. (**A**) The center of the tumor model was designated as point ‘a’ and the end of the spinal needle as point ‘b’. The displacement from point ‘a’ to point ‘b’ was calculated as ΔX and ΔY. (**B**) The displacement from the point of origin ‘a’ to point ‘b’ was determined as ΔY and ΔZ. (**C**) The center of the tumor model was designated as point ‘a’.

Intraoperative CT images were transferred to medical image viewing software (Radiant, 22.1.1 Medixant, Poznan, Poland). The center point of the tumor model with contrast enhancement and the center point of the spinal needle tip were compared using the 3D MPR function on CT imaging. First, the axes were established based on the center of the tumor model. In the sagittal view, the horizontal line through the center was set as the X-axis, and the vertical line was set as the Y-axis. Subsequently, the changes in the X- and Y-coordinates from the center to the tip of the needle were calculated. In the transverse view, the center of the tumor model was used as the origin, with the vertical line designated as the Y-axis and the horizontal line as the Z-axis. The change in the Z-coordinate from the center to the tip of the needle was then determined. The needle placement deviation was calculated as follows: √[(△X)^2^ + (△Y)^2^ + (△Z)^2^] (Figure 7).

**Figure 8:**
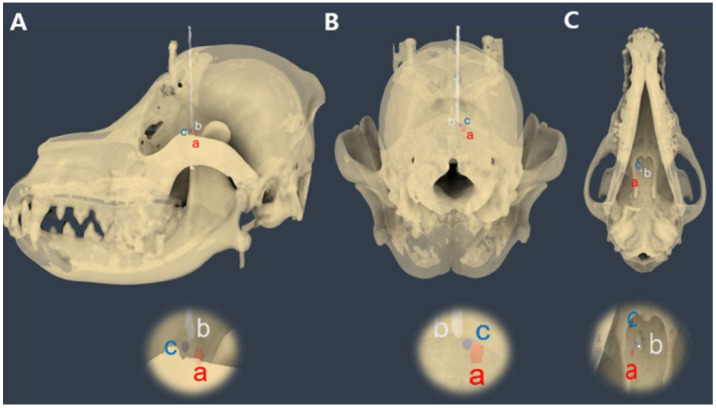
Calculation of needle placement error in the fronto-olfactory lobe after inserting a spinal needle, as assessed by the 3D volume-rendering method. (**A**) The center of the tumor model was designated as point ‘a’, the end of the spinal needle as point ‘b’, and the center of the ROI was designated as point ‘c’. (**B**) The spatial relationships of the three points in the transverse view. (**C**) The spatial relationships of the three points in the axial view. The distance between each point can be measured using the measurement function.

Intraoperative CT images were transmitted to Mimics Medical software (version 21.0; Materialize NV, Leuven, Belgium) for stereolithography (STL) file generation, similar to preoperative imaging. Segmentation was performed using appropriate thresholding techniques to isolate the skull, 3D guides, spinal needle, and tumor model. Subsequently, the STL files were transferred to computer-aided design software (Fusion 360, Autodesk, San Rafael, CA, USA). Using Fusion 360 software (Autodesk, San Francisco, CA, USA), preoperative and intraoperative STL files were fused to assess the needle placement errors. The preoperative skull was aligned with the intraoperative skull, and the endpoints of the spinal needle tip, the center point of the target ROI, and the center point of the tumor model were marked. The distance between the needle tip point and center point of the target ROI was measured. The relative orientation of the spinal needle tip point was determined using the sagittal, dorsal, and transverse views. The distance between the needle tip point and the center point of the tumor model was measured, and the positional relationships were assessed (Figure 8).

**Figure 9:**
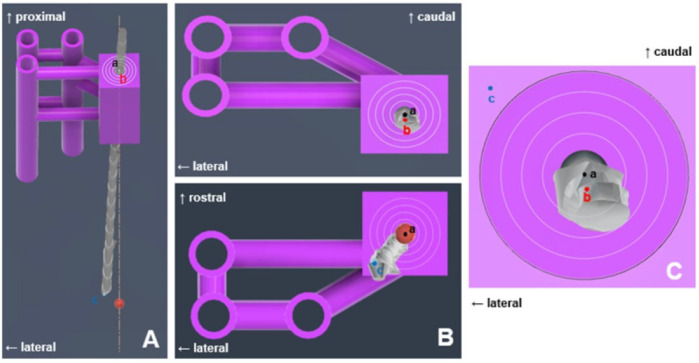
Evaluation of the needle trajectory. (**A**) Overall view of the needle trajectory. (**B**,**C**) Set the midpoint (a) of the most proximal surface of the preoperatively planned needle insertion unit and then create a circle with a diameter of 1–5 mm. Next, establish the actual center (b) where the spinal needle passes through this surface and determine the needle endpoint (c). Finally, assess the positional relationship of these points on the dorsal plane.

The entry point was defined as the point at which the needle passed through the most proximal plane of the preoperative needle guide, and the endpoint of the needle was defined as the exit point. Displacement was measured from an actual reference point. When determining the relative positions of all points, the rostral direction was designated as positive (+), the caudal direction as negative (−), the lateral value as positive (+), and the medial value as negative (−) (Figure 9).

### 2.5. Statistical Analysis

All statistical analyses were performed with SPSS (version 29.0; IBM Corp., Armonk, NY, USA). The normality of the data was assessed using the Kolmogorov–Smirnov test. Data are reported using the mean and standard deviation(SD) or median and range, depending on their normality. A paired *t*-test was performed to compare needle placement errors between the 3D volume-rendering and CT methods as the difference followed a normal distribution. The fronto-olfactory lobe and piriform lobe groups were compared using either the independent Student’s *t*-test or the non-parametric Mann−Whitney U test. A *p*-value of <0.05 was considered statistically significant.

## 3. Results

### 3.1. Needle Placement Error

Twenty spinal needle injections were administered to 10 cadavers. Two hypothetical targets were set in the brain of the cadavers: target A in the right fronto-olfactory lobe and target B in the left piriform lobe. Furthermore, the tumor model in the right fronto-olfactory lobe was designated as target A, and the tumor model in the left piriform lobe was designated as target B.

The mean needle placement error in all dogs was 2.1 mm (range, 0.73–3.53 mm). The mean needle placement error for the fronto-olfactory lobe (target A) and the piriform lobe (target B), as assessed using a 3D method, was 2.02 mm (range, 0.73–3.22 mm) and 2.18 mm (range, 1.34–3.53 mm), respectively. The independent *t*-test results indicated that there were no significant differences in needle placement errors between the fronto-olfactory lobe (target A) and the piriform lobe (target B) (*p* = 0.6).

**Figure 10:**
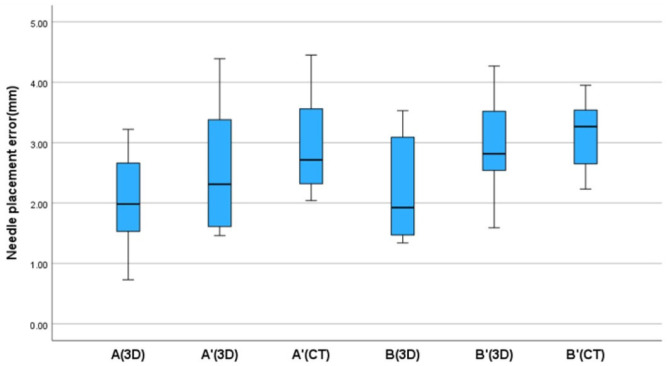
Box and whisker plots display the needle placement error of the target points in mm. A, fronto-olfactory lobe ROI; A’, tumor model of the fronto-olfactory lobe; B, piriform lobe; B’, tumor model of the piriform lobe.

The mean needle placement errors for the tumor models in target A’ and target B’, evaluated using the 3D method, were 2.54 mm (range, 1.46–4.39 mm) and 2.87 mm (range, 1.59–4.27 mm), respectively. When assessed by CT imaging, the mean needle placement errors for the tumor models in target A’ and target B’ were 2.98 mm (range, 2.04–4.45 mm) and 3.14 mm (range, 2.23–3.95 mm), respectively (Figure 10). The needle placement errors between CT and the 3D method for target A’ were found to be significantly different, as evidenced by the results of a paired *t*-test (*p* < 0.001). Similarly, a significant difference was observed for target B (*p* < 0.001) (Table 1). Independent *t*-tests did not reveal significant differences in needle placement errors between target A and A’, assessed using the 3D method (*p* = 0.159), nor between target B and B’ (*p* = 0.051). The mean errors for the ROI and tumor models were 2.55 mm (range, 1.11–4.37 mm) for target A-A’ and 3.25 mm (range, 1.94–4.2 mm) for target B-B’.

### 3.2. Trajectory Deviation

**Figure 11:**
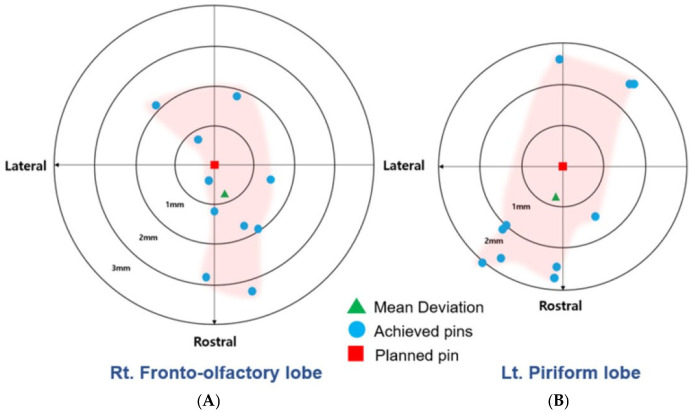
Direction and magnitude of translation of the entry point of the achieved needles (blue circles) relative to the planned needle (red square). The mean translation is represented by the green triangle. (**A**) Fronto-olfactory lobe ROI; (**B**) piriform lobe ROI.

Target A entry point translations occurred in the rostromedial direction in four, in the rostrolateral direction in three, in the caudomedial direction in one, and in the caudolateral direction in two. Mean (SD) rostrocaudal and mediolateral translations were 0.75 mm (1.72; i.e., rostral; range, −1.83–3.24) and −0.24 mm (0.94; i.e., medial; range, 1.48−1.58), respectively. Target B entry translations occurred in a rostromedial direction in one, rostrolateral direction in six, in a caudomedial direction in two, and in a caudolateral direction in one. Mean (SD) rostrocaudal and mediolateral translations were 0.80 mm (2.13; i.e., rostral; range, −2.69–2.75) and 0.28 mm (1.38; i.e., lateral; range, −1.79–2.00), respectively. An independent *t*-test indicated no significant differences in rostrocaudal (*p* = 0.95) or mediolateral (*p* = 0.32) translations between targets A and B (Figure 11).

**Figure 12:**
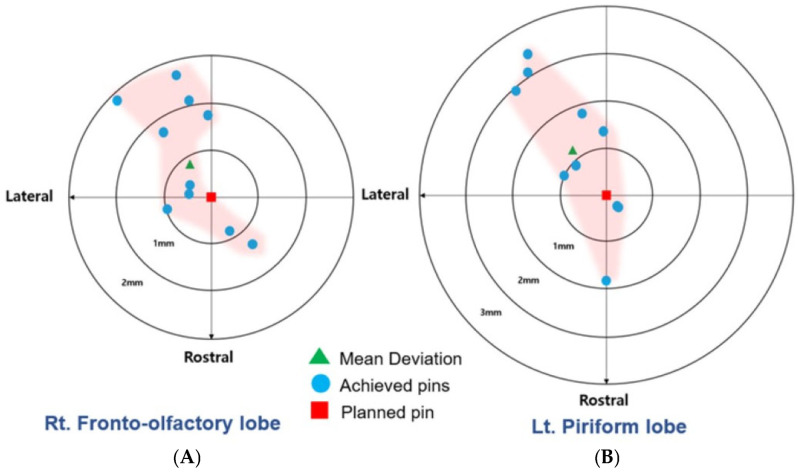
Direction and magnitude of translation of the exit point of the achieved needles (blue circles) relative to the planned needle (red square). The mean translation is represented by the green triangle. (**A**) Fronto-olfactory lobe ROI; (**B**) piriform lobe ROI.

The translations of the exit points of targets A and B were consistent and observed as follows: in the rostromedial direction in two cases, rostrolateral in one case, caudomedial in none, and caudolateral in seven cases. Mean (SD) rostrocaudal and mediolateral translations of the target A exit point were −0.83 mm (1.37; i.e., caudal; range, −2.67–1.15) and 0.52 mm (0.80; i.e., lateral, range, −0.85–2.04), respectively. Mean (SD) rostrocaudal and mediolateral translations of the target B exit point were −1.01 mm (1.53; i.e., caudal; range, −3.03–1.88) and 0.72 mm (0.85; i.e., lateral, range, −0.27–1.95), respectively. An independent *t*-test indicated that there were no significant differences in the rostrocaudal (*p* = 0.78) and mediolateral (*p* = 0.59) translations between targets A and B (Figure 12).

### 3.3. Guide Design and 3D Printing Time

The average total guide design and manufacturing time in 10 samples was 249 ± 12.43 min.

## 4. Discussion

In this study, the accuracy of a CT-based 3D printed patient-specific brain biopsy guide using a transfrontal approach was evaluated in 10 cadaver brains using 3D volume-rendering methods. Furthermore, by injecting tumor models into the fronto-olfactory and piriform lobes to simulate actual tumors, the needle placement accuracy of the CT method was compared with that of the 3D program method. The mean needle placement error was 2.01 mm (range, 0.73–3.53 mm). No significant differences were found between the target points and spinal needle placement. Previous studies on the stereotactic brain biopsy system commonly used in veterinary medicine reported mean needle placement errors ranging from 0.9 mm to 4.3 mm [15,16,21,22,23,24]. The needle placement errors were found to be from 1.5 mm up to 2.8 mm in dogs [12,19,25]. In studies using a 3D-printed biopsy device, the needle placement error ranged from 0.83 mm to 2.7 mm [7,19]. The results of this study showed trends similar to previous studies, confirming the high accuracy and precision of the 3D-psBBG. This can be attributed to the precise biopsy planning and rigid fixation provided by the 3D-psBBG.

To the best of our knowledge, this is the first study to apply 3D-psBBG in canines using an induced tumor model. Considering the high incidence of brain tumors in dogs and their relevance as brain biopsy targets, gliomas were assumed and used during the configuration of the tumor model [4,8,9]. There were two reasons for dividing the location into the fronto-olfactory and piriform lobes. First, the fronto-olfactory, parietal, and temporal lobes of the brain are common sites for gliomas, and there is a lack of research that specifically focuses on the fronto-olfactory lobe in the existing studies on 3D brain biopsy guides. Therefore, in this study, we concentrated on the fronto-olfactory lobe. Second, the addition of the piriform lobe was intended to facilitate a comparative analysis of the accuracy of the guides based on tumor depth [4,6]. The size of the tumor model was determined by reference to previous studies, setting the volume at 2 mm^3^, which was then fabricated using resin (ZMD-1000B CLEAR-SG, Zenith, Daegu, Republic of Korea) and coated with iohexol (Omnipaque^™^ 300 mg/mL) to ensure visibility on the CT scans [26].

Needle placement errors were evaluated by combining intraoperative CT with the original STL file, which had the spinal needle attached, and performed using Fusion 360 software for biopsy planning. The spherical tumor model, enhanced with contrast on CT, was visible and the distance between the center of the tumor model and the tip of the spinal needle was measured using the 3D MPR feature of RadiAnt, the open source DICOM viewer(Version 2023.1, Medixant, Poznan, Poland). Significant differences in needle placement errors were observed between the CT and 3D program methods for targets A (*p* < 0.001) and B (*p* < 0.001). This study indicates that the 3D method yields greater accuracy in calculating needle placement errors than the CT method. This is because using the DICOM viewer program with the MPR function results in lower accuracy due to potential errors in the axis setting as well as in defining the center of the tumor model and the tip of the spinal needle.

One study compared the target depth of the caudate nucleus, thalamus, and midbrain with the needle placement error and reported no statistically significant differences [15]. Two independent studies concluded that superficial lesions are biopsied with less accuracy compared to deeper lesions [16,23]. However, in this study, the mean needle target error for the fronto-olfactory lobe (target A) was 2.02 mm (range, 0.73–3.22 mm), and for the piriform lobe (target B), it was 2.18 mm (range, 1.34–3.53 mm). No significant differences were found in the needle placement errors between the two groups (*p* = 0.6), which This indicates that there was no correlation between the needle placement error and tumor depth.

A 3D patient-specific brain biopsy guide has several advantages and disadvantages. First, due to its patient-specific nature, a guide can be manufactured regardless of the type and size of an individual’s skull, allowing for biopsy procedures. In contrast, with the conventional frame-based stereotactic brain system, there are instances where some devices may not fit or align properly with the patient’s head size, posing potential issues [23]. Second, it offers ease of manipulation and does not require training for biopsy procedures compared to conventional stereotactic systems. Furthermore, it enables a precise determination of the craniectomy site and helps avoid the need for inevitable modifications to the surgical window during the procedure. As a result, research studies using 3D guide-based brain biopsy have shown high accuracy [7,18,19,27]. Third, a significant advantage of this system is its cost-effectiveness compared to traditional rigid stereotactic brain biopsy frames or contemporary neuronavigation systems [28]. Fourth, it allows one to obtain multiple samples during the biopsy procedure. The 3D guide can form multiple biopsy ports tailored to various lesions in cases of multifocal lesions. Furthermore, large lesions allow for multiple sampling of different regions of the same lesion. Although previous studies have mentioned the potential increase in side effects during multiple biopsies, it is recommended that a diverse sample is obtained, if possible, as it can improve the diagnostic yield [29,30,31].

The disadvantages are as follows. First, it requires an understanding and knowledge of the 3D software platform. Guide design and biopsy planning were performed on the software platform, requiring additional time to improve proficiency. Additionally, the design and printing processes consume time. Previous studies have reported a total duration of approximately 6.5 h, while in this study, it took 249 ± 12.43 min. Consequently, there may be delays in the immediate brain biopsy following CT imaging [19]. Second, an additional constraint of the 3D-printed brain biopsy guide is the limited trajectory option. These values were predetermined according to the design of the device. Consequently, surgeons lack the flexibility to select alternative trajectories in response to technical challenges encountered during the procedure or suboptimal specimen retrieval, as indicated by the results of smear preparation. In such cases, the surgeon must resort to an alternative method for brain biopsy such as image-guided free-hand biopsy.

In this study, there were several differences from previous research on 3D brain biopsy guides [17,19]. First, some studies used patient-specific facemasks to perform frontal lobe biopsies, where the guide contacts the skin rather than the bone [17]. In contrast, this study designed a guide that directly secures the frontal bone to minimize movement and improve safety during inner-table craniotomy of the frontal bone. In addition, a burr guide was fabricated to further increase safety. Second, previous studies lacked distinct anatomical indications for 3D guide fabrication [7,17,19]. However, in this study, the guides were manufactured based on the zygomatic process of the frontal bone, providing a directional signal for guide production. Third, for tumor insertion during transfrontal craniotomy, the osteotomy was performed in a triangular shape to closely mimic a diamond-shaped bone flap, allowing for accurate injection of the tumor model.

In this study, the needle placement error was assessed, and the trajectory of the needle was examined. By connecting the entry and exit points of the spinal needle, deviations from the planned trajectory were identified [32,33]. Twenty spinal needles were injected with respect to the center of the target, occurring rostromedially in four cases, rostrolaterally in two, caudomedially in none, and caudolaterally in 14 cases, with the mean exit translation mirroring the needle deviation. The predominant caudolateral insertion was attributed to the gravitational sag of the needle in the craniomedial direction, despite aligning the patient’s head horizontally on the table and positioning the guide as planned during intraoperative CT. This error can be mitigated in clinical practice if the surgeon manually supports the biopsy needle used during the procedure.

Potential sources of needle placement errors are as follows. First, the design of the 3D-psBBG in the Fusion 360 software may have introduced errors that affected the biopsy planning process. Second, shrinkage during 3D printing could have altered the size of the guide, potentially causing it to not properly seat the skull as planned, resulting in errors. Third, inaccuracies may have arisen during the calculation of needle placement errors due to the inability to perfectly align preoperative and intraoperative CT images in the image fusion process. Fourth, errors between the tumor model and the spinal needle may have occurred because the tumor model did not reach the intended target during insertion.

This study had some limitations. First, all 3D-psBBGs in this study were manufactured based on CT imaging. Although CT offers better details of the selected bone surface and subsequent guide development and design, MRI offers a better contrast resolution for lesion identification. Consequently, to improve the accuracy of needle placement in clinical cases, performing additional MRI is necessary [17,34]. Second, while cerebrospinal fluid (CSF) collection and MRI are essential in veterinary diagnostics, they do not definitively distinguish between inflammatory and neoplastic brain lesions. Consequently, histopathological analysis using brain biopsies is essential. However, this study used spinal needles instead of specialized brain biopsy needles in normal cadaveric brains solely to evaluate needle accuracy, highlighting the need for additional clinical research to validate the findings [35]. Third, one of the most common complications of brain biopsy is intracranial or intratumoral hemorrhage originating from the biopsy track. Other complications include the leakage of CSF due to penetration of the lateral ventricle and gas into the soft tissues surrounding the calvarium. However, in this cadaveric study, it was not possible to verify the occurrence of these complications due to the study’s nature, as it was conducted on cadavers and lacked postoperative evaluation [12].

To create a hole in the internal table of the frontal bone, the application of an internal burr unit poses challenges due to the limited surgical space, which could lead to difficulty in manipulation and the risk of brain damage. Therefore, more studies and the development of additional internal burr units are warranted to address this issue.

## 5. Conclusions

The CT-based 3D printed patient-specific brain biopsy guide using the transfrontal approach allowed for accurate needle placement within 3.53 mm of the intended target in each procedure, leading to the successful procedure in 10 cadaveric canine subjects. Furthermore, when comparing needle deviations resulting from tumor model insertion using the CT and 3D methods, we found that the 3D approach was more accurate. Finally, we determined that there were no significant differences in needle accuracy relative to tumor depth. Future studies with larger populations and more clinical cases are warranted to determine the flexibility of the system with various target accuracies.

## Figures and Tables

**Table 1:** Needle placement error of targets A’ and B’ using the 3D and CT methods.

Type		Needle Placement Error			t(*p*)
		N	M	SD	
Target A’	3D	10	2.54	1.00	5.678 (<0.001) ***
	CT	10	2.98	0.79	
Target B’	3D	10	2.87	0.77	6.674 (<0.001) ***
	CT	10	3.14	0.56	

*** *p* < 0.001.

## Data Availability

The raw data supporting the conclusions of this article will be made available by the authors on request.

## Supplementary Materials

The following supporting information can be downloaded at: https://www.mdpi.com/article/10.3390/ani14213163/s1. Video S1: Brain Biopsy Procedure Using a 3D-Printed Guide in Dogs.
